# Supplementary material for: The landscape of metabolic pathway dependencies in cancer cell lines
Source: PLoS Comput Biol. 2021 Apr 19;17(4):e1008942. doi: 10.1371/journal.pcbi.1008942 (PMC8084347; doi:10.1371/journal.pcbi.1008942)

We would like to thank the Reviewers for their comments about our manuscript. We appreciate the Reviewers' constructive criticisms, their close attention to detail, and their appreciation that our approach demonstrates the importance of considering metabolic pathways over individual metabolic genes. Thanks to the Reviewers' comments, our manuscript has been significantly strengthened. Below, we offer a point-by-point response to the Reviewers' comments, which are listed in black text. Our responses are in purple text.

Reviewer #1: Manuscript title: The landscape of metabolic pathway dependencies in cancer cell lines

Summary:

The manuscript by Joly et al., describes an integration of genetic dependency data (DepMap CRISPR/CAS screens), gene expression data (RNAseq), and pharmacological drug sensitivity information (DepMap PRISM) on CCLE cell lines and provides a means for metabolic pathway-level interpretation. Their approach identifies novel and previously known links between metabolic pathway activity (expression levels) in a given cell line, and essentiality of genes in various metabolic pathways as well as sensitivity of cell lines to drugs targeting various metabolic pathways. Overall, they developed a useful analysis pipeline for interpretation of multi-level treatment data on cancer cell lines and provide valuable insights into future attempts for drug discovery, personalized medicine, and combination therapies. Overall, I think it is a solid work that fits well the scope and standards of the journal PLOS Computational Biology. I believe this manuscript is suitable for publication subject to the following revisions.

Major points:

1- Figure 3 legend is inconsistent with the text. Line 215 mistakenly states that folate concentration is 4 times higher in RPMI than DMEM, while the reverse is true and consistent with the main text (line 226). Also, line 216 in the legend mistakenly states dependency on Folate is higher in DMEM, while the reverse is true and consistent with the figure and main text.

We thank the reviewer for pointing out these errors. They are indeed correct that DMEM has a higher concentration of folate than RPMI and that dependency on folate is higher in RPMI. We have changed the Figure 3 legend to correct these errors.

2- Figure 3, right color bar, shows Folate twice, with different corresponding dependency values. Please correct.

The two pathways in question, folate biosynthesis and one-carbon pool by folate, both use folate, but do so in different ways. The folate biosynthesis pathway ***produces*** folate (i.e., folate is an output) whereas the one-carbon pool by folate pathway ***consumes*** folate (i.e., folate is an input). Therefore, we chose to annotate the right color bar on Figure 3 with folate for both pathways. However, this was not clearly explained in the figure legend. We have added the following text to the Figure 3 legend to make this clearer: "Folate is shown twice because it is both the product of Folate Biosynthesis and the input to One-Carbon Pool by Folate."

3- Line 232: The authors attribute the difference seen between RPMI and DMEM in dependency to Oxidative phosphorylation to the fact that RPMI and DMEM contain 150 μM and 0 μM aspartate, respectively. They should add a plausible explanation as to why this difference is not also reflected in the dependency to Aspartate, Alanine, Glutamate metabolic pathway which also involves aspartate.

The reviewer is correct that there appears to be a discrepancy between the dependencies on OxPhos and the Alanine, Aspartate, and Glutamate metabolic pathway. We posit that the inclusion of Alanine and Glutamate metabolism genes in the “Aspartate, Alanine, and Glutamate” pathway may be a confounding factor. Unfortunately, KEGG does not have a pathway or module for aspartate metabolism. To avoid bias in our pathway selection, we chose to not include any hand curated gene sets in this study. However, because this was an interesting question, we first attempted to answer it by curating a pseudo-“Aspartate Metabolism” pathway. Specifically, we made a gene set of all the enzymes that directly connect to aspartate within the Alanine, Aspartate, and Glutamate metabolism gene set (i.e., ASNS, ASRGL1, ASS1, ADSS1, ADSS2, ASPA, NAT8L, CAD, IL4I1, GOT1L1, GOT1, GOT2, and DDO). We ran this gene set through our analysis pipeline and found that neither cell lines in RPMI nor those in DMEM exhibited strong dependence on the pseudo-aspartate metabolism pathway (weighted average NESs: RPMI = -0.096, DMEM = -0.572). We next attempted to query an "Aspartate Biosynthesis" pathway. Unfortunately, there are only two human genes listed in KEGG that synthesize but do not consume aspartate (i.e., ASRGL1 and ASPA with bi-directional enzymes like GOT1/GOT2 excluded). With only two genes, it is not possible to obtain sufficient statistical confidence to see differences in dependencies between RPMI and DMEM like those that we see in Folate Biosynthesis. Regardless, we agree with the reviewer that there is a discrepancy, and so we have added the following sentence in the text:

"Interestingly, we did not observe a strong difference in dependency on Aspartate, Alanine, and Glutamate metabolism (hsa00250) between RPMI and DMEM despite the difference in aspartate concentrations. We posit that the inclusion of alanine and glutamate metabolism genes may be a confounding factor for analyzing aspartate dependency."

4- The authors need to add some discussion/clarification about the effects of media composition on dependencies. Many instances shown in Figure 3 are not consistent with the overall conclusions of this section (lines 234-236) claiming media composition affects pathway dependency. For instance, Glutathione, Histidine, and Phenylalanine are example of reagents with considerable differences in fold abundance between the two media conditions, but no apparent effect on relative dependencies. Overall, I think in order to be able to claim a general effect from media composition (or culture mode) on dependencies, a robust statistical method such as ANOVA should be used and appropriate p-values should be reported.

We thank the reviewer for the suggestion to provide a statistical justification for our claim that media composition affects metabolic pathway dependency. To address this suggestion, we divided metabolic pathways into two groups: 1) pathways that contain metabolites with differential abundance in cell culture media (e.g., Glycolysis-Gluconeogenesis (glucose), Folate biosynthesis (folate)); and 2) pathways that do not contain metabolites with differential abundance in cell culture media (e.g., fatty acid metabolism and fructose and mannose metabolism). We then performed a paired Mann-Whitney-U test comparing the metabolic pathway dependency in RPMI and DMEM for both groups of pathways.

We found that pathways that contain differentially abundant metabolites in cell culture media exhibited significantly different pathway essentialities in DMEM and RPMI ($p = 3.9x10^{-4}$). In contrast, pathways that do not contain differentially abundant cell culture media metabolites did not exhibit significantly different pathway dependencies ($p = 0.545$). We have added this information to the Results section of the manuscript as well as the legend of Figure 3.

Minor points:

1- The authors used ssGSEA, which is a rank-based enrichment method, for inferring metabolic pathway activities based on expression levels of individual genes. The choice of this approach over alternative methods such as normalized weighted average expression should be justified either by direct comparison or at least by a logical explanation.

The reviewer is correct that normalized weighted average expression (NWAS) could potentially serve as a better metric of metabolic pathway activity than ssGSEA. To directly compare NWAS with ssGSEA, we re-ran our pipeline using NWAS to analyze dependency on the Pentose Phosphate Pathway. We chose the Pentose Phosphate Pathway for this comparison because several enzymes are shared between pathways (e.g., PRKL, PFKM, and PKFP are present in both Glycolysis-Gluconeogenesis and Pentose Phosphate Pathway gene sets) As shown in Supp. Fig. 6, we found broad agreement between the metabolic pathway dependencies when using either NWAS or ssGSEA for both Adherent RPMI and Adherent DMEM cell lines (Spearman r of 0.606 and 0.691, respectively). This data supports that either ssGSEA or NWAS can be used to infer metabolic pathway activity for this pipeline.

2- Throughout the manuscript, the authors only consider 69 of a total of 91 metabolic pathways defined by KEGG. The rationale behind this choice is not clear.

The reviewer is correct that there are 91 metabolic pathways within KEGG. However, in our analysis, we did not consider pathways that a) are not expressed in human metabolism (e.g. “Limonene and Pinene Degradation”, which has no listing for “hsa” (homo sapiens)); or b) contained less than five genes in the CCLE gene expression data (e.g., D-Glutamine and D-glutamate metabolism). After removing these pathways, we were left with 61 pathways. We have added the following sentence to the Methods to clarify this for the reader:

> “We removed pathways that either 1) are not expressed in human metabolism or 2) contain less than five genes in the CCLE gene expression data set.”.

3- Line 153-154 is vague. Instead of using the word “association”, the authors should use “enrichment” (GSEA FDR<0.05).

Thank you for the suggestion - we have made this change!

4- Line 194: The authors raise an interesting question about whether there might be a link between a metabolic pathway's essentiality and its own activity level. They report that of the 69 metabolic pathways queried, 36 had a negative NES and 33 had a positive NES. From this, they conclude that there is no general rule regarding pathway expression and essentiality. A question that bears asking is whether any meaningful differences could be detected between the pathways that fall into the above two group (corresponding to the 2 peaks shown in supp. Figure 3A). The authors should at least include the names of the pathways in each of the two groups in a supplementary table rather than only reporting the numbers 33 and 36 to facilitate further investigation beyond what is currently stated in the manuscript.

We agree with the author and have now added these results in Supp. Table 2. To better demonstrate the data, we have also rescaled Supp. Fig. 4B to have the same x- and y-axis scales. Furthermore, we did find that there was a single gene set that exhibited significant 'self-dependency': Riboflavin Metabolism (hsa00740). In both DMEM and RPMI, Riboflavin Metabolism exhibited significant negative Genetic PDEA NES values, indicating that when Riboflavin Metabolism expression is low, the dependency on Riboflavin Metabolism gene sets increases. We have now added the following sentence to the manuscript:

"While there was not general agreement between the self-dependencies, one gene set did exhibit significant Genetic PDEA NES for both Adherent RPMI and Adherent DMEM cells. Specifically, Riboflavin Metabolism (hsa00740) exhibited significant negative Genetic PDEA NES, indicating that when Riboflavin Metabolism activity is low, the dependency on Riboflavin Metabolism genes increases."

5- Line 244: Please describe how this AUC is measured and why IC-50 is not used instead.

The area-under-the-curve (AUC) is measured by fitting four-parameter logistic curves to viability values for each compound and cell line as described in Corsello et al. 2020, *Nature Cancer*. For the reviewer, a screenshot of the methods section from Corsello et al. 2020 is included below. To clarify this for the reader, we have added the following sentence to the Methods section:

"Here, the AUC represents the dose dependent effect of a drug on cell growth, calculated by fitting a four-parameter logistic curve to viability values for each compound and cell line, with a lower AUC representing a stronger response to drug [21]."

Dose–response analysis. Dose–response relationships were obtained by fitting four-parameter logistic curves to viability values for each compound and cell line using the R package drc (version 3.0-1). Following the practice of Smirnov et al.[44], the upper asymptote of the logistic curves was fixed at 1 and the viability values were fitted as a function of drug concentration according to:

$$V(c) = E_\infty + \frac{1 - E_\infty}{1 + e^{HS(c - EC_{50})}}$$

where all concentrations are in the natural logarithm scale. IC_{50} values were defined as the concentration c at which $V(c) = 0.5$. Additionally, the dose–response area under the curve (AUC) was calculated using the normalized integral:

$$AUC = \frac{\int_{c_{min}}^{c_{max}} V(c) dc}{c_{max} - c_{min}}$$

This formulation puts AUC values on a scale between 0 and 1 for curves with lower asymptotes <1, where lower AUC values indicate increased sensitivity to treatment.

6- Line 276: Typo: “activity can be associated with anti-cancer drug sensitivity”

Thank you for pointing this out, we have corrected the typo.

7- I am curious to see if the method introduced in the present manuscript could be used to test the experimental findings by Gao et al. (Dietary methionine influences therapy in mouse cancer models and alters human metabolism Nature volume 572, pages397–401(2019)) showing that methionine deprivation has a synergistic anti-cancer effect with 5-Fluorouracil therapy? I think this could potentially add an interesting validation to the present manuscript.

We thank the reviewer for the suggestion. We unfortunately know of no way to query the effects of methionine deprivation from our data. However, in an attempt to relate methionine metabolism to 5-fluorouracil therapy, we tested how sensitivity to 5-fluorouracil was correlated with the activity of two pathway modules related to methionine metabolism: 1) Methionine salvage pathway (hsa_M00034) and 2) Methionine degradation (hsa_M00035). Please note that these modules were not present in our original analysis because we had included KEGG pathways but not KEGG modules. In cancer cell lines cultured in DMEM, we found that 5-fluorouracil showed a weak correlation with decreased methionine salvage pathway activity (rho = -0.339, p = 0.0066, p-adj = 0.659). In other words, when the methionine salvage pathway activity was low, cells were more sensitive to 5-FU. However, as noted, this correlation was not significant following multiple hypothesis correction. Meanwhile, the methionine degradation module (hsa_M00035) showed no correlation with 5-fluorouracil response (rho = -0.153, p = 0.23, p-adj = 0.89). In cancer cell lines cultured in RPMI, neither the methionine salvage pathway nor the methionine degradation pathway showed a significant correlation with sensitivity to 5-fluorouracil (rho = 0.04, p-adj = 0.968 and rho = 0.17, p-adj=0.22 respectively).

Reviewer #2: OVERVIEW:
In the manuscript, "The landscape of metabolic pathway dependencies in cancer cell lines", the authors describe a novel pipeline which attempts to draw a correlation between CRISPR-Cas9 loss-of-function essentiality screens, pharmacological, and gene expression data. Indeed, the authors were able to find correlations. The pipeline is poorly described and it is very difficult for the reader to find crucial details about the analysis. Also the need for the pipeline is not clearly delineated by the authors in the Introduction, discussion, methods or results. The authors have written a lengthy and redundant manuscript for example methods section of simulation studies is basically same as what they wrote in Results section. The authors apply their pipeline to sample data to show its sensitivity. However, no comparison between the sample data and real data is available. The pipeline described by the author is unclear to me; however, I feel like the analysis presented in the paper still needs more clarity and fleshing out. Further, the pipeline makes use of a lot of data only to recapitulate existing knowledge and very little new findings. The state of the manuscript still feels preliminary to me, in terms of both analysis and presentation, and leaves a lot to be desired. Keeping all this in mind, I recommend a major revision.

MAJOR CONCERNS:
1. The authors literally gave two paragraphs of background in the introduction before jumping to what they did in the paper. We often write Introductions in the paper to establish the relevance of the work and give the readers some context on where authors are coming from. Metabolic pathway dependencies in cancer are studied often and should have plenty of information for the authors to discuss. Please rewrite the entire section with some relevant examples as discussed in the results section. I would make some suggestions but I would review rather than write the paper for them.

We thank the Reviewer for the suggestion. We have added additional background to the Introduction of the manuscript, and we hope that these paragraphs will provide the reader with more context for our study. In addition, we would like to highlight that our study tests metabolic *pathway* dependencies rather than metabolic *gene* dependencies (e.g., enolase 2) or *metabolite* dependencies (e.g. glucose, methionine). We believe that the focus on pathways rather than individual genes or metabolites adds systems biology relevance to our study, and we have attempted to emphasize this point in the Introduction.

2. The shape of the gene expression distributions of different cell lines may be significantly different. How similar are the gene expression distributions of simulated data from the real data (of the 16643 genes) for each of the 300 cell lines? Further, some of the cell lines are similar which the real data (both gene dependency and expression) will reflect.

We thank the Reviewer for the suggestion to look at the gene expression distribution of the cell lines. In our initial submission, we simulated data using a normal distribution with mean (μ) of 0 and standard deviation (σ) of 1. However, upon reviewing the data, we found that simulated data from a normal distribution with $\sigma = 0.5$ better reflected the gene expression data used in our analysis. To demonstrate this, we have included a version of Supplementary Figure 1 (below) with the $\mu = 0$ and $\sigma = 1$ distribution included. (The $\mu = 0$ and $\sigma = 1$ distribution is omitted from Supp. Fig. 1 of the manuscript.) Accordingly, we have updated our simulation studies to analyze simulated gene expression data from a normal distribution with $\mu = 0$ and $\sigma = 0.5$ rather than $\mu = 0$ and $\sigma = 1$ (Fig. 1 B,C and Supp. Fig.1).

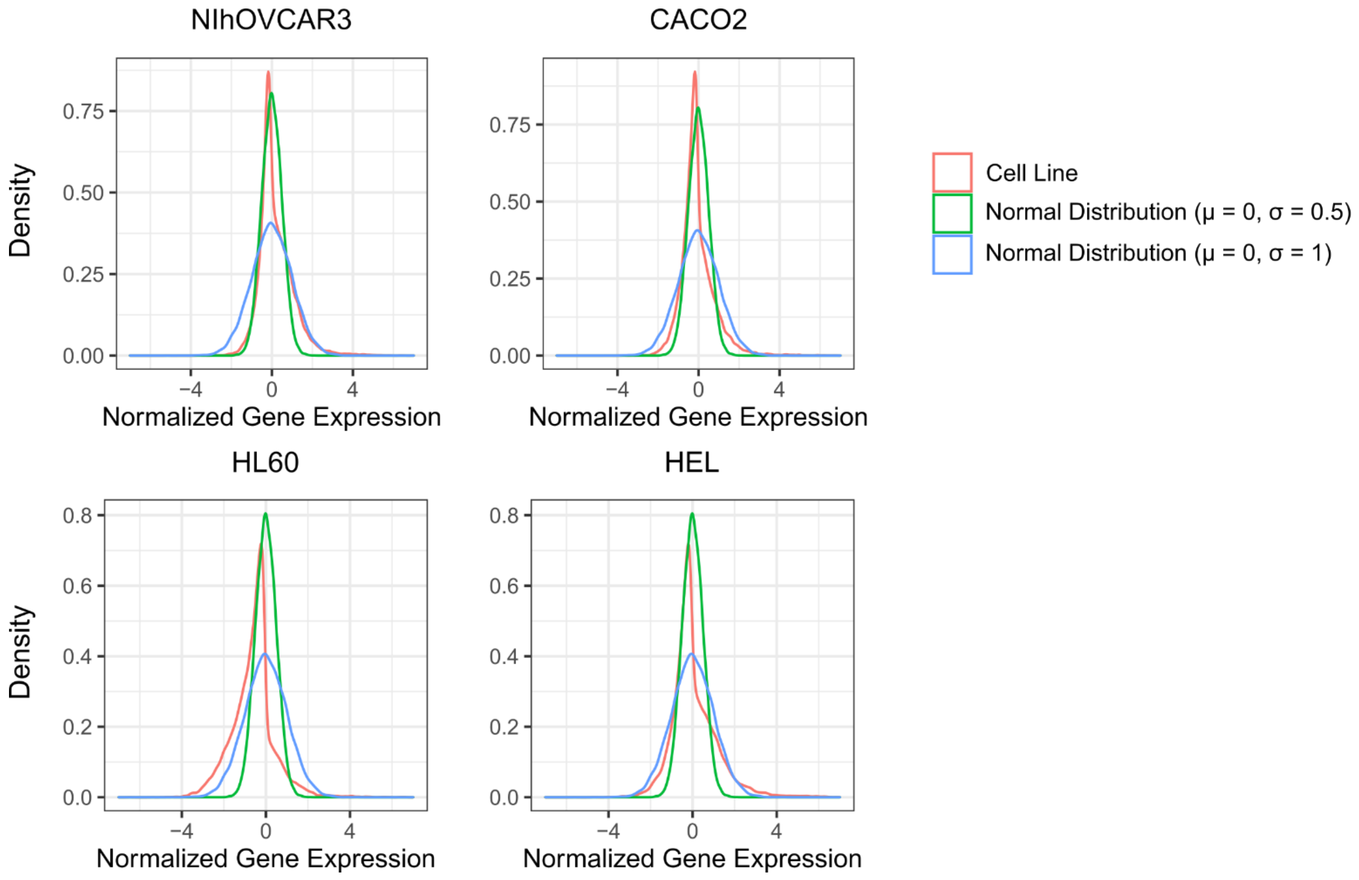

Supporting Figure 1. A normal distribution reflects gene expression values. Gene expression data was taken from the cancer cell line encyclopedia and scaled and centered within culture type (adherent or suspension) and culture medium (DMEM or RPMI). Four cell lines were chosen at random and their gene expression profiles were compared to a normal distribution with a mean of 0 and a standard deviation of 1 (blue) or 0.5 (green).

Do the authors introduce some "meaning" in their simulated data that their pipeline is supposed to catch? How do the authors ensure the pipeline will catch meaningful signals? Also, Could the authors provide a better way to understand 1B and 1C? What is the sensitivity of Genetic PDEA conveying? What does it mean? In line 118, what is a significant results here? The method can be sensitve, but can it capture accurate results? How do the authors know this? Perhaps some benchmarking and comparison of this simulated data would be useful for the reader to understand. The authors may have this in the supplementary information.

Thank you for these questions. We designed our simulated data with exactly these questions in mind. First, yes, the simulated data does contain meaning. Specifically, to create a signal in the simulated data, we created a synthetic gene set consisting of 25 genes that were perturbed with an expression gradient of size X (i.e., the “signal”) relative to the background genes. First, for all 300 simulated cell lines, the values of the 16,618 background genes were drawn from a normal distribution with $\mu = 0$ and $\sigma = 0.5$ (i.e., the “noise”). Then, for cell line #1, the values for the 25 genes in the synthetic gene set were randomly drawn from a normal distribution distribution with $\mu = -X$, $\sigma = 0.5$. Similarly, the values for the 25 genes in the synthetic gene set for cell line #300 were randomly drawn

from a normal distribution with μ = +X, σ = 0.5. For cell lines #2-299, the values of the 25 genes in the synthetic gene set were drawn from normal distributions with sequentially increasing values μ from -X to X. For example, if X = 1, the values of the 25 genes in the synthetic gene set were drawn from normal distributions with:

- cell line #1: μ = -1, σ = 0.5
- cell line #2: μ = -0.9933, σ = 0.5
- cell line #3: μ = -0.9866, σ = 0.5
- ...
- cell line #150: μ = -0.0033, σ = 0.5
- cell line #151: μ = +0.0033, σ = 0.5
- ...
- cell line #299: μ = +0.9866, σ = 0.5
- cell line #300: μ = +1, σ = 0.5

In this way, we create a *gradient* of perturbed gene expression values for the synthetic gene set across the 300 cell lines, with the perturbation being most negative for cell line #1 and most positive for cell line #300. We also created a similar perturbation *gradient* in the gene essentiality values for the 25 genes in the synthetic gene set across all 300 cell lines. Next, the gene expression data were subjected to ssGSEA to calculate the synthetic gene set's normalized enrichment score (NES) (i.e., pathway activity) in each cell line, and the ssNES values were correlated with the gene essentiality values for all 16,643 genes. Using the rank list of correlations between pathway activity and gene essentiality, we then calculated the genetic PDEA normalized enrichment score and the associated p- and q-values.

To test the sensitivity and accuracy of our approach, we varied the value of X from 0 to 0.5 for both gene expression ($X_{expression}$) and gene essentiality ($X_{essentiality}$). For each combination of $X_{expression}$ and $X_{essentiality}$, we ran the simulation 50 times. To summarize the results, we then plotted heat maps of the percentage of replicates for which 1) the FDR-corrected p-values for the correlation coefficients between ssNES and gene essentiality for the 25 genes in the synthetic gene set were less than $1x10^{-4}$ (Figure 1B); and 2) the genetic PDEA q-value was less than 0.01, respectively (Figure 1C).

We believe that this approach tests both the sensitivity and the accuracy of our computational pipeline. For example, when both $X_{essentiality}$ and $X_{expression}$ were small (0.05), we observed ~0% of significant results. Thus, the pipeline does not identify false positives at a significant rate. In contrast, when $X_{essentiality}$ and $X_{expression}$ are both larger than ~0.2, we found that the 100% of the 50 replicates exhibited q-value < 0.01 (Figure 1C). Thus, our pipeline identifies perturbed pathways with good sensitivity. Taken together, this simulated data thus benchmarks the performance of the pipeline against data where we have explicitly introduced meaning through the parameters $X_{essentiality}$ and $X_{expression}$.

3. What does the metabolic pathway activity mean here? In Figure 1, what are the dimensions of sub-figure A1. The pipeline description is not clear.

We thank the reviewer for pointing out that this was unclear. Here, metabolic pathway activity means metabolic flux. Because we do not have measurements of metabolic flux (i.e., stable isotope tracing), we inferred metabolic pathway activity (i.e., flux) from the gene expression data using ssGSEA. Although this is an indirect means of assessing metabolic flux, we and others have shown

that this is a reasonable approximation for activity of metabolic pathways (PMIDs 32692836, 31434891 25456139, 22713172).

In sub-Figure 1, panel A1, the x-axis represents the list of 16,643 genes ranked by expression, and the y-axis represents the running enrichment score (as done in GSEA). The dimensions are similar in Figure 1 panel A4. We have labeled these axes to clarify.

4. If the authors are using DepMap dependency data which tells you the essentiality of the gene, why is there a need summarize the data at the level of pathway so early in the analysis. The authors could be loosing information by doing this. Can't we correlate the genes themselves and then finding the pathways with high positive or negative correlation coefficients.

We agree that there is information to be gained from looking at individual gene correlations. However, for this study, we chose to focus on pathways rather than individual genes because 1) metabolic pathways consist of multiple enzymes which collectively regulate metabolic flux; and 2) others have already explored the dependency of cancer cells on individual metabolic genes (e.g., PMID: 31039782). Regardless, the enrichment analysis we used in this study does identify the genes driving the enrichment for each pathway, and we have included these gene lists in Supporting Table 1 (see column “Leading Edge Genes”). Because we recognize that this information was not abundantly clear to the reader, we have added the following sentence to the manuscript “Leading edge genes for each significant pathway dependency are listed in Supp. Table 1.”.

5. Analysis in Fig 2, did that involve extraction before or after Genetic PDEA? Also how were genes belonging to multiple pathway treated?

The results in Figure 2 are from the Genetic PDEA analysis. The normalized enrichment scores and the false discovery rates are from the last step in the pipeline drawn in Figure 1A. If genes belonged to multiple pathways, we included them in each pathway (i.e., no weighting factors were included for genes that belong to multiple pathways). However, as described in our response to Reviewer #1’s Minor Point 1, we found that an alternative metric that does account for genes belonging to multiple pathways (Normalize Weighted Average Expression) gave similar results to our ssGSEA method.

6. At line 201, by context, do the authors mean environment (media)? Also, it should be obvious, no? that the pathway dependencies were influenced by media? A metabolic gene that is essential in one media doesn't need to be essential in another. However, it would be more curious if this were true for significant number of non-metabolic genes and pathways. Also, Could the authors show how much influence media has over the NES and correlation coefficients?

At the previous line 201 (current line 197), the word “context” refers to the effects that metabolic pathway *activity* has on metabolic pathway *essentiality*. For example, in Figure 2B we show that increased *activity* of the One-carbon pool by folate pathway strongly correlates with increased *dependency* on the folate biosynthesis pathway. However, as shown in Figure 2A, folate biosynthesis pathway is not universally essential in all cancer cell lines. To make this clearer for the reader, we have

modified the sentence in question: “Rather, these results indicate that metabolic pathway dependency is highly context specific such that metabolic pathway activity influences metabolic pathway essentiality”

7. The way the whole analysis is done, the authors continue to integrate any new data they find. However, it would be nice if authors could validate some of their analysis. Alternatively, present new targets or novel unexplored pathway vulnerabilities, for e.g. using analysis in Fig 6. As of now, it seems like a method that is very expensive that needed transcriptomic data, gene essentiality data, and then pharmacological data, to produce known findings.

We agree that it would be very expensive to generate transcriptomic data, gene essentiality data, and pharmacological data for hundreds of cell lines. However, by building on a plethora of publicly available data, our computational pipeline did in fact generate novel findings. For example, we demonstrated dependency on the TCA cycle is high when pentose phosphate pathway activity but not glycolysis is high (Supp. Fig. 3). To emphasize these novel but experimentally untested findings, we have added this sentence to the manuscript: “This new finding suggests that the diversion of glucose from glycolysis to the pentose phosphate pathway may confer increased dependency on the TCA Cycle.” Finally, we agree that experimental validation of our findings is a worthy goal. However, we feel that experimental validation is beyond the scope of this computational manuscript. We hope that our computational manuscript will spur the cancer metabolism community to begin testing our predictions.

MINOR CONCERNS:

1. Line 101: Should that ssNES?

Thank you for the suggestion. We have made this change.

2. Line 133: "Then, a synthetic gene set of 25 genes was perturbed..." For better visualization of the perturbation, could authors also say how much does the value of each gene possibly change. Also what is X and its value?

X represents the value of the perturbation for each gene in the synthetic gene set. Please see our more detailed description above in response to Major Concern 2.

3. Something doesn't see right with the sentence in line 159. Are authors trying to say "...dependencies are clustered together based on pathway activities more than activities are clustered based on dependencies..."? How? It is not clear from just looking at the figure 2A or Supp Fig 1?

We agree that the original sentence was confusing. We have revised this sentence to be clearer:

“We next clustered Genetic PDEA NES values across all pathway activities (columns) and pathway dependencies (rows) (Fig. 2A & Supp. Fig 2A) and found that related pathways

clustered together on the x-axis (pathway activity) but not on the y-axis (pathway dependency). This suggests that pathway activity but not pathway dependency is similar within a group of related pathways."

In addition, we have added colored pathway categories to Figure 2A. These pathway categories were taken directly from KEGG. With this addition, it will hopefully be clearer to the reader that clustering is stronger on the x-axis (pathway activity) than it is on the y-axis (pathway dependency) for related metabolic pathways (e.g., glycan metabolism, carbohydrate metabolism).

Reviewer #3: The present study is intended to provide a comprehensive characterization of metabolic pathway vulnerabilities in cancer cell lines. The authors describe computational value scores of metabolic pathway activity and calculate associations between such scores and sensitivity to clinically approved drugs. They report that metabolic pathway dependencies are highly context-specific and that cancer cells are vulnerable to inhibition of one metabolic pathway only in conjunction with another specific metabolic pathway. As an example, they argue that their approach implemented for Pentose Phosphate Pathway may serve to identify which patients that respond to antifolate chemotherapies. Overall, while the study has potential value for future applications, the authors should provide at least some experimental evidence, for example test the effects of combinations predicted by the computational model. Such experiments should be done in various media and O2 concentrations to recapitulate tumor microenvironment heterogeneity. In absence of any experiment, it is difficult to judge the value of such computational predictions.

We thank the Reviewer for recognizing the “potential value for future applications” of our results. We agree that experimental validation of our predictions is a worthy goal. However, we feel that experimental validation is beyond the scope of this computational manuscript. In addition, we note that our methodology did identify several relationships which are supported by published evidence, such as a relationship between the pathways “One-Carbon Pool by Folate” and “TCA Cycle” (Fig. 2C) (PMID: 30613765). We hope that our computational manuscript will spur the cancer metabolism community to begin testing our predictions.

More specific concerns highlighted below:

1. Starting at line 264, the authors state that they “found a strong association between decreased Core Glycolysis (hsa_M00001) pathway activity and increased sensitivity to AZD8931, an inhibitor of EGFR and ERBB2. This should be a good test case in principle, but still unclear to me how it would be done exactly. For example treat a collection of cells with a combination of AZD8931 and inhibitors of various glycolytic enzymes? (how many and on which exact enzymes?). I presume more than one, otherwise it would undermine the foundation of the paper (the importance of considering the pathway rather than individual genes).

We agree with the Reviewer that the relationship between decreased Core Glycolysis and increased sensitivity to the EGFR inhibitor AZD8931 should be a good test case for our methodology. As noted above, however, we feel that experimental validation of this prediction is beyond the scope of this computational manuscript. That said, one potential method for testing the hypothesis that decreased glycolytic flux increases sensitivity to AZD8931 would be to test for synergy between glycolytic inhibitors and AZD8931, perhaps using a method like the Combination Index (PMID: 20068163).

2. This reviewer remains unsure about the meaning (and usefulness) of metabolic pathway score/value as described. For example, in the case of cholesterol biosynthesis, I understand the essentiality score for HMGCR, or sensitivity scores for statins. When it comes to the entire pathway, my understanding is that the calculation is based on every component of the pathway. However, within any given pathway only a few steps are druggable and/or are rate limiting for the respective metabolic flux. To shut down an entire pathway one need only block a few select steps.

The reviewer is correct that our calculation of metabolic pathway activity is based on every component of the metabolic pathway and that this approach could lead to erroneous estimates of metabolic pathway activity. However, we note that we have previously found a GSEA-based approach to accurately capture metabolic pathway activity including the shift towards glycolysis in hypoxia (Joly et al., *Bioinformatics*, PMID 32692836) and the inhibition of nucleotide synthesis in senescent cells (Delfarah et al, *JBC*, PMID 31138644). This has been corroborated by others as well, such as the use of GSEA to identify metabolic features for immune cells within the tumor microenvironment (Xiao et al., *Nat Comm*, PMID PMC6704063). As such, we address this weakness in the penultimate paragraph of the Discussion:

"Another potential weakness of our study is that we rely on the inference of metabolic pathway activity from gene expression data. Gene expression, however, does not always accurately reflect cellular metabolism. First, proteomic studies have shown that protein expression does not always correlate with gene expression [52]. Second, metabolic enzyme activity can be regulated by post-translational modifications [53,54]. By using gene expression data, we have not accounted for these factors, and as such our analysis may not reflect pathway activity at the metabolic flux level. We expect that expanding recent efforts to characterize metabolite abundance [33] and metabolite flux [55] in panels of cancer cell lines will improve our ability to identify metabolic pathway vulnerabilities by providing better measures of metabolic pathway activity."

3. The reported correlations are based on datasets generated using CRISPR screens. The authors should consider recapitulating the analysis using the RNAi screen data (also available on DepMap). There are many cases of discrepancies between shRNA and CRISPR based screens (Avana, Demeter scores). In absence of any experimental approach, it would only be prudent to generate computational models using both types of data.

We agree with the reviewer that it would be great to include other types of data to examine metabolic pathway dependencies. There are, however, many discrepancies between RNAi screen data and CRISPR screen data. Importantly, the DEMETER2 scores do not account for differences in DNA copy number alterations. Therefore, we chose to use CERES scores that does computationally correct for copy number alterations (PMID: 29083409). Furthermore, off-target effects of RNAi have been extensively characterized, including a very interesting recent example that led to misidentification of the mechanism of action of an anticancer drug (PMID: 31511426).

Nevertheless, we did want to try to integrate other data to validate our results. To achieve this, we took CRISPR dependency data from the Sanger Institute and ran our Genetic PDEA pipeline. Notably, the data from the Sanger Institute used different experimental protocols, reagents, and a different QC pipeline but was still corrected for copy number effects to calculate a CERES score. A recent study has shown that robust biomarkers of gene dependency found in one data set are recovered in the other (PMID: 31862961). Therefore, we tested the reproducibility of metabolic pathway vulnerabilities using our Genetic PDEA pipeline. To do this, we first calculated metabolic pathway activity using CCLE gene expression data (Fig. 1A, Step 1), then correlated the metabolic pathway activity ssNES values to either CERES scores from DepMap or from the Sanger institute (Fig. 1A, Step 2), and then compared the results of Genetic PDEA from both data sets (Fig. 1A, Steps 3 and 4). Because this comparison relies on the same metabolic pathway activity data, we combined the

DepMap and Sanger p-values using the harmonic mean p-value (HMP) which is appropriate for combining dependent statistical tests while controlling the family-wise error rate (FWER) (PMID: 30610179). Despite significant differences in experimental protocols and reagents in CRISPR-Cas9 screening, this approach demonstrated that the Genetic PDEA found in the DepMap data set were broad reproduced in the Sanger data set:

Cell Type	Number of significant metabolic pathway vulnerabilities		Percent of DepMap pathways significant in combined result
	DepMap data set alone (FDR < 0.05)	Combined DepMap and Sanger data sets (HMP < 0.05)	
Adherent RPMI	224	215	96%
Adherent DMEM	186	167	90%

We believe this broad agreement demonstrates that our method is reproducible between two large pan-cancer gene dependency data sets from different institutes. We have added this information in the manuscript as:

1. Supporting Figure 5: Broad agreement for Genetic PDEA between two large CRISPR-Cas9 gene dependency data sets.
2. Supplementary Table 3: Comparison of Genetic PDEA for gene dependency data sets between the Broad and Sanger Institutes

And have added the following sentences:

“To examine whether these findings were reproducible, we analyzed data from another large-scale pan-cancer CRISPR-Cas9 gene dependency data set (Sanger Institute) [35] using the Genetic PDEA pipeline. Since the underlying gene expression profiles were derived from the CCLE for both Sanger and DepMap Genetic PDEAs, we combined statistical tests using the harmonic mean p-value (HMP) which combines dependent statistical tests while controlling the family-wise error rate (FWER) [36]. Applying an HMP threshold of 0.05, we found that 96% and 90% of significant results in the DepMap Genetic PDEA were recapitulated in the combined DepMap and Sanger Genetic PDEA results for Adherent RPMI and Adherent DMEM cells, respectively (Supp. Fig. 5 and Supp. Table 3).”

The figures depict CCLE collection as a “nondescript cloud” without further analyses based on lineages, key mutations, etc. Any correlations between the pathway score and individual scores for rate limiting enzymes?

The reviewer is correct that while we have accounted for environmental factors (e.g. culture type, culture medium), we did not account for lineage effects in our analysis. In our initial submission, we did not pursue this analysis because the number of available cell lines for each lineage is often quite low (usually less than 20, see table below). Thus, we felt that we would lack statistical power to justify lineage-specific metabolic vulnerabilities.

Cell lineage	Num. cell lines	Media
NSCLC	58	RPMI
melanoma	29	RPMI
glioma	25	DMEM
gastric_adenocarcinoma	19	RPMI
esophagus_squamous	19	RPMI
ovary_adenocarcinoma	17	RPMI
exocrine	17	RPMI
multiple_myeloma	17	RPMI
upper_aerodigestive_squamous	16	DMEM
colorectal_adenocarcinoma	15	RPMI

However, in response to this question, we sought to justify our intuition by testing how our Genetic PDEA pipeline would perform with a smaller number of cell lines. We thus ran a simulation study of Genetic PDEA (like in Fig. 1) with n = 30 cell lines rather than n = 300. As shown below, we found that sensitivity was strongly decreased with only 30 cell lines. Although we do agree that these analyses should be run on lineage-specific data sets (especially since lineage can significantly affect drug response, e.g., PMID: 31292550), we feel that we do not currently have the statistical power to justify lineage-specific analysis in the current data set.

n = 30 cell lines

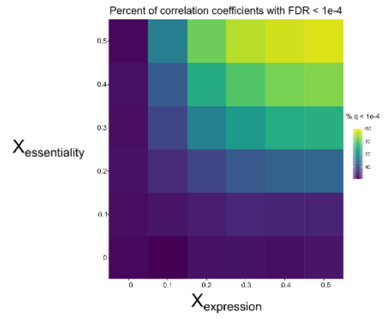

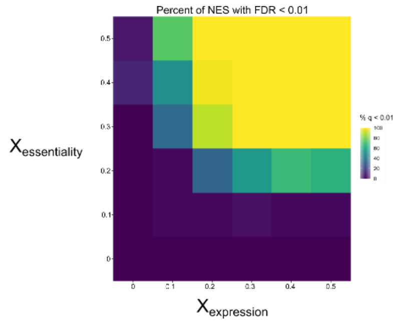

n = 300 cell lines (Fig. 1)

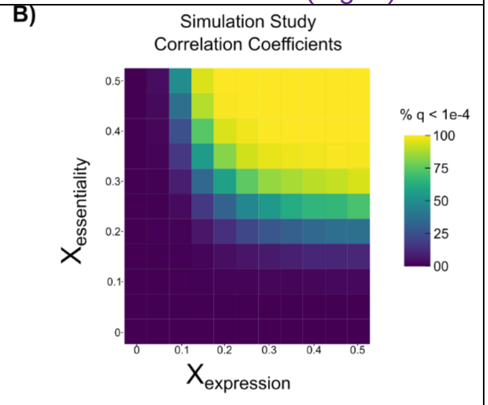

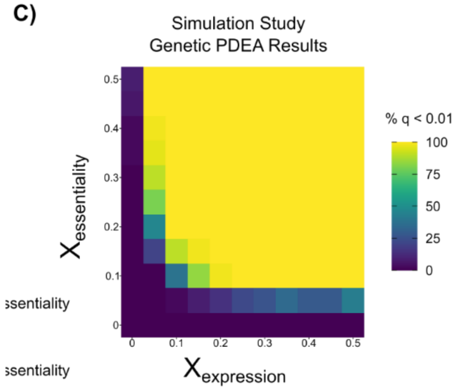

Supplement: Attachment — Submitted filename: Response to Reviewers FINAL.pdf [file pcbi.1008942.s017.pdf]
